# A Self-Assembling Peptide as a Model for Detection of Colorectal Cancer

**DOI:** 10.3390/gels8120770

**Published:** 2022-11-25

**Authors:** Yuan Wan, Ruyue Luo, Jialei Chen, Xinyi Luo, Guicen Liu, Di Su, Na Lu, Qichen Liu, Zhongli Luo

**Affiliations:** 1 College of Basic Medical Sciences, Molecular Medicine and Cancer Research Center, Chongqing Medical University, Chongqing 400016, China; 2 Department of Medicine, Northwestern University Feinberg School of Medicine, Chicago, IL 60611, USA; 3 College of Pediatrics, Chongqing Medical University, Chongqing 400016, China

**Keywords:** self-assembling peptide, colorectal cancer, accurate molecular detection, three-dimensional culture

## Abstract

Patient-derived organoid (PDO) models have been widely used in precision medicine. The inability to standardize organoid creation in pre-clinical models has become apparent. The common mouse-derived extracellular matrix can no longer meet the requirements for the establishment of PDO models. Therefore, in order to develop effective methods for 3D cultures of organoids, we designed a self-assembling peptide, namely DRF3, which can be self-assembled into ordered fibrous scaffold structures. Here, we used the co-assembly of self-assembling peptide (SAP) and collagen type I, fibronectin, and laminin (SAP-Matrix) to co-simulate the extracellular matrix, which significantly reduced the culture time of PDO, improved the culture efficiency, and increased the self-assembly ability of cells. Compared with the results from the 2D cell line, the PDO showed a more significant expression of cancer-related genes. During organoid self-assembly, the expression of cancer-related genes is increased. These findings provide a theoretical basis for the establishment of precision molecular modeling platforms in the future.

## 1. Introduction

Colorectal cancer has one of the highest morbidity and mortality rates worldwide, mainly due to metastatic and drug resistance [1,2]. Traditional colorectal cancer research is mainly based on tumor cell lines and cell line-transplanted tumor models. These models lack the microenvironment and heterogeneity of clinical tumors, and cannot truly reflect the development mechanism and drug response of clinical colorectal cancer.

For a long time, the most common method of human disease exploration has been cell and animal models [3,4]. As an extremely important tool and carrier, animal models have many advantages: they avoid the risk of human experiments and overcome the inconvenience caused by the long incubation period and low incidence of some diseases [5,6]. As one of the most widely used research models, cell models are the basic tools in many life science research fields, including genetics [7,8], stem cell science [9,10,11], immunology [12,13] and so on. These two models are widely used but are also subject to many limitations. Due to species differences, animal models are quite different from human beings in physiological structure, tissue and organ functions [14,15], etc., and so it is difficult to truly and accurately reflect human physiological changes. Because of the lack of connection between tissues and organs, the cell model does not directly simulate human body function [16]. Therefore, organoids have gradually become a research hotspot.

In recent years, the rise of the organoid technique has made it possible to realize the simultaneous cultivation of cancer tissue and normal tissue from the same patient [17,18]. It can be better used to evaluate the efficacy and safety of drugs [19,20], carry out the large-scale screening of drugs [21,22], and provide help in formulating a physical treatment plan for patients with different diseases [23,24]. Tumor organoid models have overcome the many shortcomings of traditional research models, and have been increasingly widely used in tumor development [25,26], metastasis mechanism [27,28], drug screening [29,30], individualized therapy [31,32] and other fields, becoming a hot topic in tumor research.

Tumor organoids can simulate the tumor microenvironment [33], preserve the interaction between cells [34], and reflect the somatic mutations [35] and biological characteristics of the tumor [36]. These characteristics make tumor organoids an ideal model for precision therapy and basic research. When the tumor develops to a detectable size, the unique regional microenvironment surrounding the tumor cells is generated, which has a significant impact on the biological characteristics of the tumor cells. These characteristics cannot be simulated in a two-dimensional culture. Therefore, 3D culture of tumor cells is considered as an ideal model for oncology research.

At present, existing methods for tumor organoid culture rely entirely on poorly defined animal-derived matrices, such as Matrigel [37,38]. However, Matrigel contains various murine-derived growth factors that may produce spurious and physiologically unrelated pleiotropic effects on tumor organoids. In this study, a self-assembling peptide hydrogel and a human-derived matrix were combined to construct a new culture platform for a three-dimensional culture of an organoid source of colorectal cancer. Based on in vitro 3D cell culture technology, a colorectal cancer organoid model can be constructed in a 3D system, which can better maintain tumor heterogeneity and retain the influence of the tumor local complex microenvironment, laying the foundations for the establishment of an individualized accurate molecular model platform for colorectal cancer.

## 2. Results and Discussion

### 2.1. Congo Red/Aniline Blue Staining Analysis

**Figure 1:**
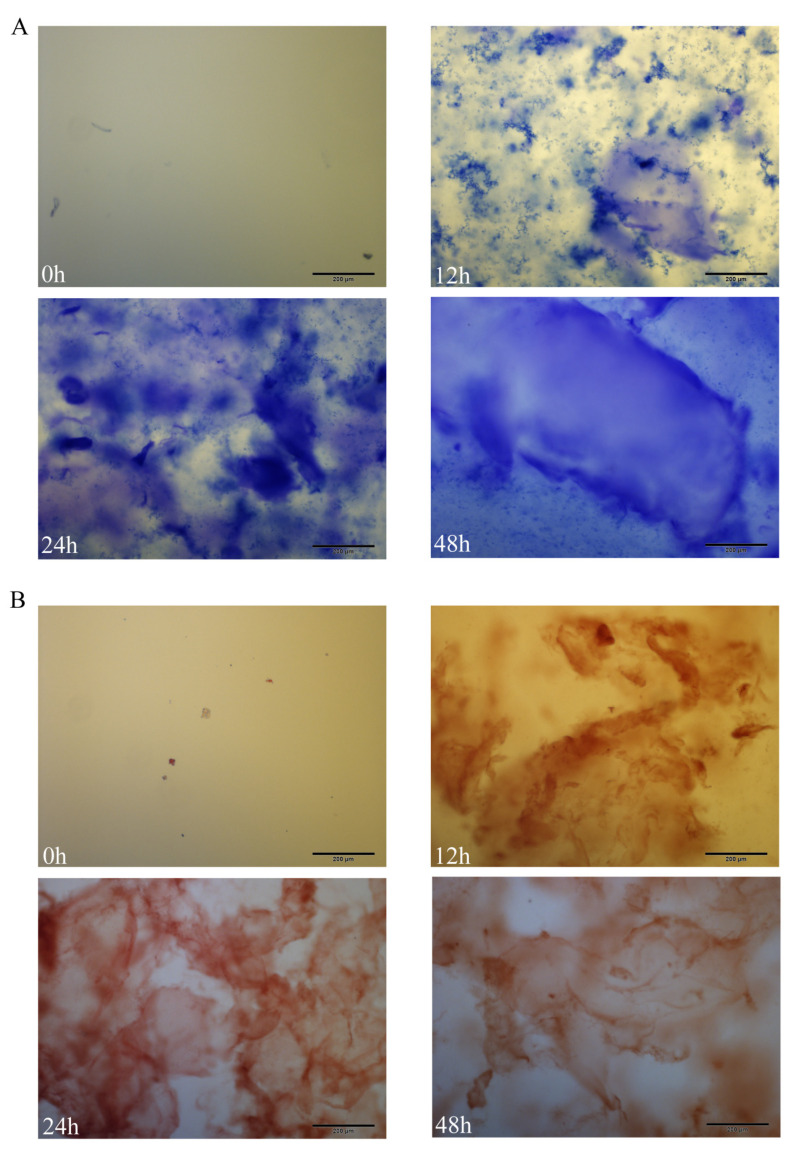
Congo red/aniline blue staining analysis. (**A**) Aniline blue staining results of DRF3 at 0, 12, 24 and 48 h; (**B**) Congo red staining results of DRF3 at 0, 12, 24 and 48 h.

At 0 h, no obvious morphology was observed under the microscope; at 12 h, DRF3 was observed to be dispersed in a thin film morphology; at 24 h, DRF3 was observed to have a dense thin-film morphology under the microscope; at 48 h, it was observed that DRF3 presented a stable large range with a lamellar membrane morphology (Figure 1).

### 2.2. Physicochemical Characterization of DRF3

#### 2.2.1. AFM Analysis of DRF3

**Figure 2:**
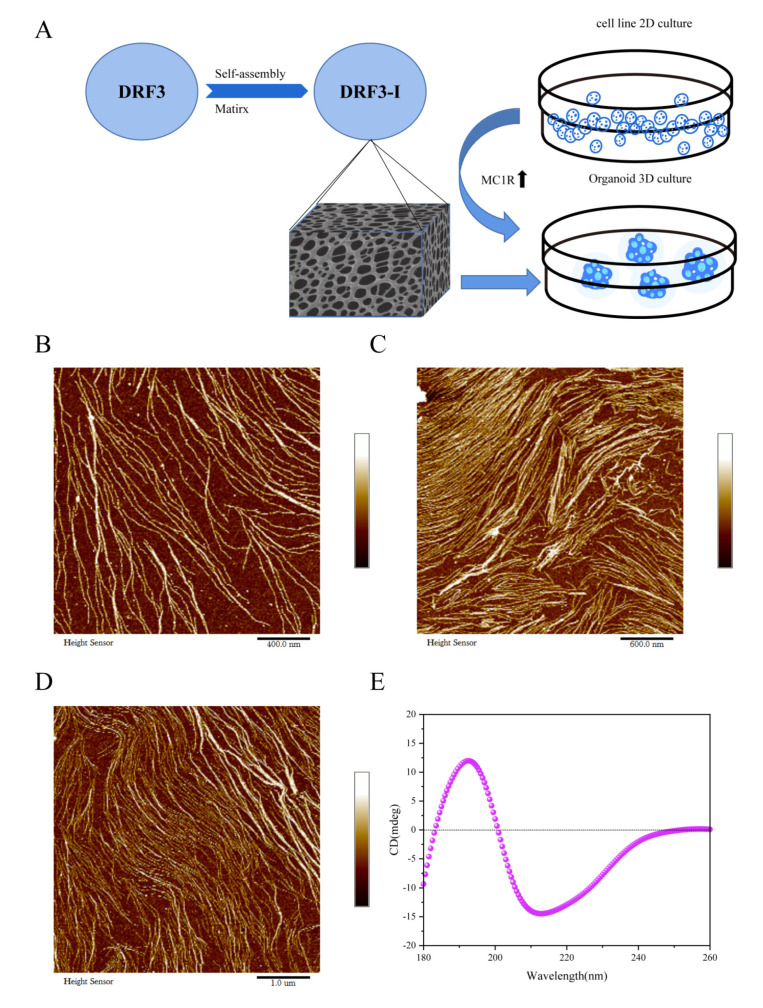
Microscopic morphology and secondary structure analysis of DRF3. (**A**) Schematic illustration of detection in the 3D culture with SAP-Matrix compared to the 2D cell line. (**B**–**D**) AFM morphology of DRF3 at 400 μm, 600 μm and 1 μm. The fiber width of DRF3 ranges from 7 to 24 nm, with an average width of 11.67 nm. (**E**) Secondary structure of DRF3. DRF3 has a positive peak around 195 and a negative peak around 216.

AFM results show that DRF3 can form ordered nanofiber scaffolds after self-assembly. The fiber width of DRF3 ranges from 7 to 24 nm, with an average width of 11.67 nm (Figure 2A).

#### 2.2.2. Secondary Structure Analysis of DRF3

Circular dichroism results indicate that DRF3 has a positive peak around 195 and a negative peak around 216 (Figure 2B), revealing that DRF3 has β-sheet secondary structure.

### 2.3. Growth Curves of Organoids in SAP-Matrix and Matrix

**Figure 3:**
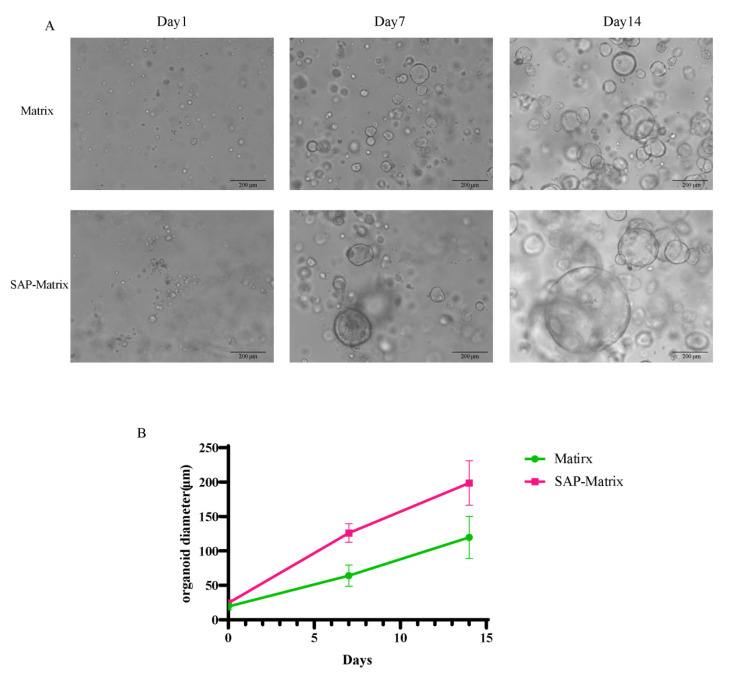
Morphology and growth curve analysis of organoids in the SAP-Matrix and Matrix. (**A**) Microscopic morphology of organoids of Caco-2 with SAP-Matrix and Matrix on day 1, 7, and 14; (**B**) growth curve trend of organoids of Caco-2 with SAP-Matrix and Matrix in 14 days.

The SAP-Matrix group and the Matrix group both enable CACO-2 to be self-assembled into organoids. On day 7, the SAP-Matrix group formed larger-diameter and a larger quantity of organoids, and on day 14, the effect of the SAP-Matrix group on the formed organoids was more significant (Figure 3).

### 2.4. Growth Curves of Organoids

**Figure 4:**
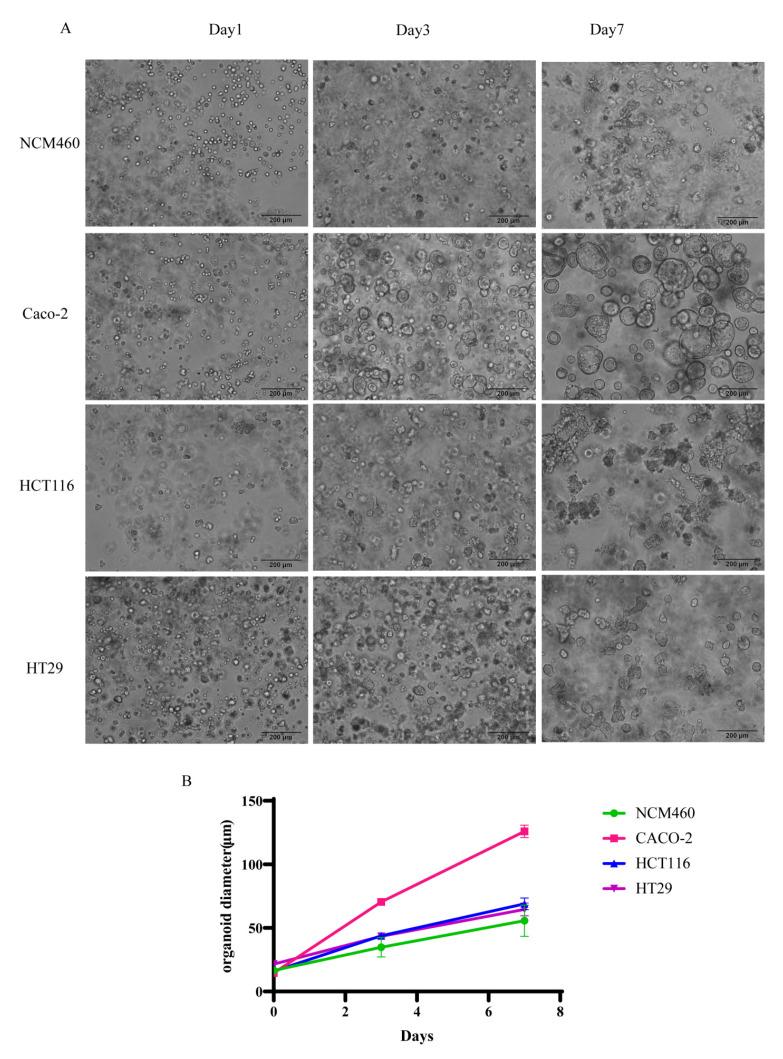
Morphology and growth curve analysis of organoids in the SAP-Matrix. (**A**) Microscopic morphology of organoids of NCM460, Caco-2, HCT116, and HT29 on day 1, 3, and 7; (**B**) growth curve trend of organoids of NCM460, Caco-2, HCT116, and HT29 in 7 days.

On day 1, the cells in all the groups were scattered. On day 3, NCM460, Caco-2, HCT116 and HT29 cells self-assembled into a small-diameter vacuolar organoid morphology. Compared with other groups of cells, Caco-2 cells had the largest organoid diameter and clearer structure. On day 7, the diameters of organoids increased in all groups, and those of Caco-2 had the largest diameter and clearest structure (Figure 4).

### 2.5. IHC Analysis of Organoids

**Figure 5:**
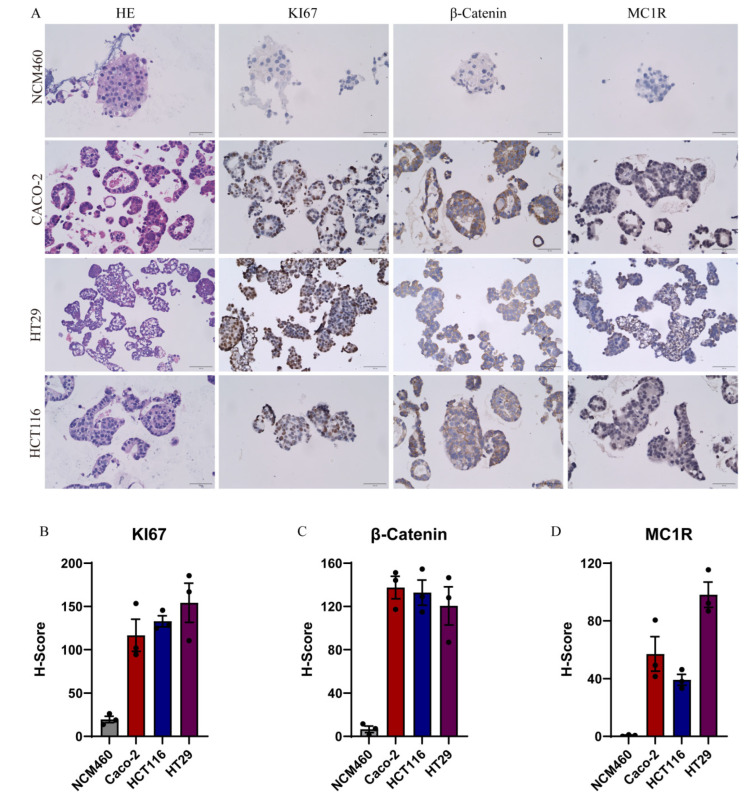
IHC and H-Score analysis of organoids. (**A**) HE, KI67, β-Catenin and MCIR staining results of organoids of NCM460, Caco-2, HCT116, HT29; (**B**−**D**) H-score analysis of KI67, β-catenin and MCIR of organoids of NCM460, Caco-2, HCT116, HT29. The black dots represent the values of the sample.

The HE results show that NCM460, Caco-2, HCT116 and HT29 self-assembled to form organoids (Figure 5A). The immunohistochemical results of β-Catenin and KI67 confirmed the high expression of β-catenin and KI67 in NCM460, Caco-2, HCT116 and HT29 (Figure 5A,B).

### 2.6. Western Blot and qPCR

#### 2.6.1. Western Blot

**Figure 6:**
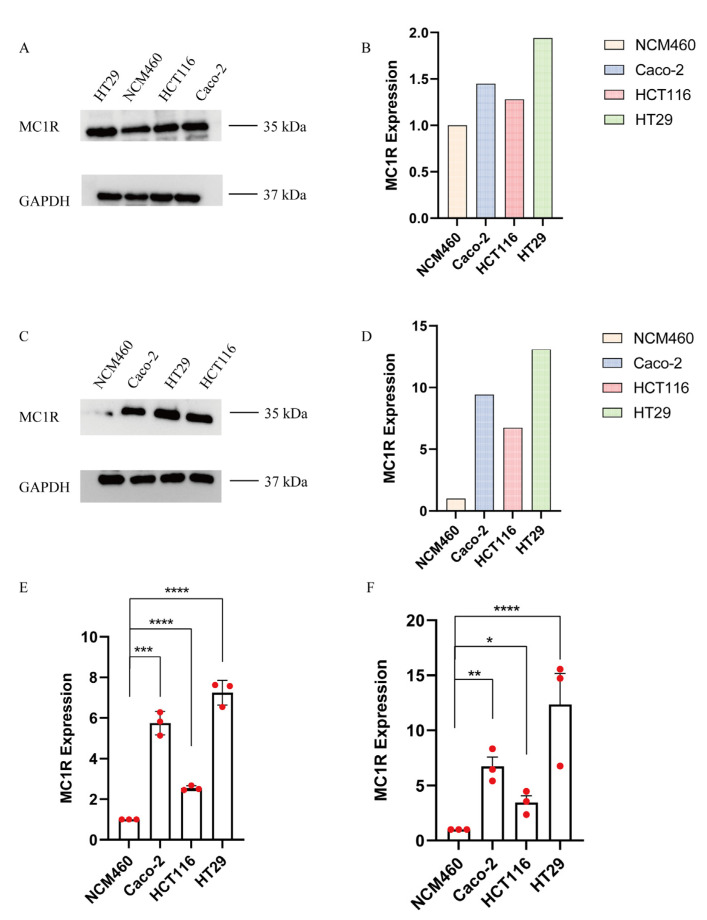
The expression of MC1R was detected by WB and qPCR. (**A**,**B**) Protein expression of MCIR in 2D cell lines; (**C**,**D**) protein expression of MCIR in organoids; (**E**) expression of MCIR in 2D cell lines by qPCR; (**F**) expression of MCIR in Organoids by qPCR (the results were repeated three times, shown as mean ± SEM; * significant differences at *p* < 0.05; ** significant differences at *p* < 0.01; *** significant differences at *p* < 0.001; **** significant differences at *p* < 0.0001). The red dots represent the values of the sample.

In 2D cell lines, the expression of MC1R in Caco-2, HCT116 and HT29 increased 1.5–2-fold compared with NCM460 (Figure 6A,B). In organoids, the expression of MC1R was increased 8–13-fold compared with NCM460 (Figure 6C,D).

#### 2.6.2. qPCR

In 2D cell lines, MC1R showed a 2.5–8-fold increase in gene expression compared with NCM460 (Figure 6E). In organoids, MC1R showed a 5–15-fold increase in gene expression compared with NCM460 (Figure 6F).

### 2.7. Discussion

In our previous preclinical organoid screening model, we found that the self-assembly ability of different samples was uneven under the same culture conditions, which greatly reduced the stability of patient screening. Therefore, we planned to construct a new extracellular matrix for simulating the extracellular matrix more accurately. The 3D tumor model based on a matrix refers to multicellular masses (or cell aggregates) obtained by 3D culture of tumor cells inoculated with matrices as scaffolds in vitro, which have certain self-renewal and self-assembly capabilities and can demonstrate the structure and function of tumors in vivo to a certain extent. Biomaterials, for example PHAs [39], have been used for multiple cell and tissue engineering applications. Compared to PHAs, the self-assembling peptide DRF3 is composed of amino acids and has better biocompatibility and a stronger scaffold structure and have been widely used to reconstruct normal stem cell niches for modeling neurogenesis [40], osteogenesis [41], and chondrogenesis [42]. Therefore, the hydrogel DRF3 combined with collagen type I, fibronectin, laminin was used to culture organoids. Here, we use the self-assembling peptide hydrogel DRF3 to mimic key features of the in vivo microenvironment, allowing organoids to be cultured in vitro. The co-assembled DRF3 provides an ordered fiber microenvironment. The establishment of this stent with stable composition and standardization lays a stable foundation for research into precision medicine, new drug development, preclinical models and disease development

Recent studies have shown that MC1R is involved in the initiation and progression of colorectal cancer [43]. Our results show that MC1R was highly expressed in 2D colon cancer cell lines and organoids, and the trend was more obvious in organoids. This indicates that organoids cultured in DRF3 environment can be closer to the in vivo environment, and the expression of related genes will be more obvious. This provides the rationale for precision medicine and drug screening. At present, most of the known experiments are based on 2D models, such as the development of new targets and gene knockout technology. The establishment of this model verified that there is still a certain gap between 2D culture and 3D culture. Therefore, the development of 3D culture technology in the future is interesting, not only limited to the development of a new extracellular matrix, but also exploring the accuracy of the model and the reliability of the experiment.

## 3. Conclusions

In this paper, a self-assembling peptide DRF3 was designed to self-assemble into ordered nanofiber scaffold structures. When used in combination with a matrix, the microenvironment in vivo can be simulated more accurately. SAP-Matrix, which contains human-derived matrix to mimic the extracellular matrix, will go further in the field of precision medicine and reveals great potential in providing standardized drug screening models for patients. This precision molecular model platform also demonstrates that when cancer cells are in the process of self-assembly, the expression of markers will be disparate, indicating that the existing cell line-derived verification models are not particularly accurate. Therefore, the application of organoids as a precision molecular detection model could become a rising star in the future.

## 4. Materials and Methods

### 4.1. Materials

Collagen type I (Corning (New York, NY, USA)), fibronectin (R&D Systems (Shanghai, China)), laminin (R&D Systems (Shanghai, China)), T25 breathable cell culture flask (LABSELECT (Hangzhou, China)), 1 × PBS buffer (Biosharp (Anhui, China)), DMEM medium (Gibco (Shanghai, China)), and bovine serum albumin V BSA (Albumin Bovine serum) (BioFrox (Einhausen, Germany)). Six-well plates (Nest (Jiangsu, China)). Trypsin (Hyclone (Logan, UT, USA)). Antibodies (Abcam Company (Cambridgeshire, UK)). The organoid medium (Chongqing Kingbiotech Co., Ltd. (Chongqing, China)).

### 4.2. Micromorphology of DRF3

The sequence of DRF3 is Ac-(Arg Leu Asp Ile Lys Val Glu Phe)_2_-CONH_2_.

#### 4.2.1. Assemble DRF3

The 10 mg/mL self-assembled peptide stock solution was prepared by mixing 1 mL of PBS with 10 mg of DRF3 lyophilized powder.

#### 4.2.2. Congo Red/Aniline Blue Staining

Congo red/aniline blue dye was mixed 1:1 with DRF3 at 0 h, 12 h, 24 h and 48 h of DRF3 self-assembly, and the morphology of DRF3 was observed under a microscope.

#### 4.2.3. DRF3-I Preparation

PA-Matrix was prepared using DRF3 (1 mg/mL), collagen type I (1.5 mg/mL), fibronectin (50 μg/mL), and lamining (50 μg/mL). Ultrasonic removal of air bubbles was performed.

### 4.3. Microscopic Morphology Observation of DRF3

We used a JASCO J-815 Spectrometer (JASCO J-815, Japan) to detect the secondary structure of DRF3.

We used AFM (Brooke Multimode8, USA) to detect the surface topography and structure information of DRF3.

### 4.4. Cell Culture

#### 4.4.1. Two-Dimensional Cell Line Culture

NCM460, Caco-2, HCT116, and HT29 were purchased from BNCC and cultured in DMEM medium containing 10% FBS in a 37 °C incubator with 5% CO_2_.

#### 4.4.2. Three-Dimensional Culture in SAP-Matrix

The cells in the logarithmic phase of growth were harvested, the cell concentration was adjusted to 1 × 10^5^ cells, and the cells were resuspended with 1 mL of SAP-Matrix. Cells were seeded in 6-well plates. Two milliliters of organoid medium was added to each well, cultured at 37 °C incubator with 5% CO_2_.

### 4.5. RNA Isolation and RT-PCR

#### 4.5.1. RNA Isolation

RNA of PDO was isolated from DRF3-I using an RNA extraction kit. DRF3-I was first removed by trypsin and incubated at 37 °C for 20 min.

#### 4.5.2. qRT-PCR

##### Primers

GAPDH Forward primer: CCGCATCTTCTTTTGCGTCG;

GAPDH Reverse primer: TTCACCTTCCCCATGGTGTC;

MC1R Forward primer: TCCGTCTGCTCCAATGACTG;

MC1R Reverse primer: TTCACATCCCAGCTGACGAG.

##### qRT-PCR

The quality of RNA was quantified using a Nano Drop 2000 spectrophotometer. One microgram of RNA was reversely transcribed into cDNA using a reverse transcription kit. qPCR was performed using SYBR Green rapid mixing of hypoxic reagents and PCR reactions were performed using the Quantstudio real-time PCR system.

### 4.6. Western Blotting

Organoids were lysed in RIPA lysate and phosphatase inhibitors were added. The organoid concentration was measured using the BCA protein concentration Assay Kit. A 20 µg protein sample from each organoid sample was used for SDS-PAGE electrophoresis. The film was then placed on a transmembrane with transmembrane paper and then blocked overnight at 4 °C in blocking solution. The cells were then incubated with MCIR primary antibody (1:3000) or GAPDH primary antibody (1:2000) for 2 h at room temperature, then were washed three times and incubate with IgG anti-rabbit secondary antibody (1:5000) for 1.5 h at room temperature. After incubation with the ECL kit, imaging was performed with a Western blot imager.

### 4.7. IHC Analysis of Organoids

Organoids were harvested and embedded in 2% AGAR. They were fixed with 4% paraformaldehyde, immersed in the xylene solution for 20 min, dehydrated and embedded in paraffin blocks. Paraffin sections were cut and pasted onto slides, heated at 100 °C for 20 min and then overnight at 37 °C. They were then immersed in the xylene solution for 10 min, and then immersed in 75% ethanol for 10 min. Slides were stained for H&E. IHC staining was then performed. The slides were blocked with 1%BSA for 1 h and incubated with primary antibodies such as human-specific antibodies KI67, β-Catenin, and MC1R for 1 h, and then the antibodies were visualized using the DAB peroxidase substrate kit. The results were analyzed using Image J.

### 4.8. Data Statistics

All the experiments in this paper were repeated three or more times. The results are shown as mean ± SEM. GraphPad Prism 8.4.3 was used to compare the experimental results between the two groups (two independent-sample *t*-tests and one-way ANOVA). ‘*’ means significant differences at *p* < 0.05; ‘**’ means significant differences at *p* < 0.01; ‘***’ means significant differences at *p* < 0.001; ‘****’ means significant differences at *p* < 0.0001.

## Data Availability

The data presented in this study are available in the article.
